# Mapping the Landscape of Care Providers’ Quality Assurance Approaches for AI in Diagnostic Imaging

**DOI:** 10.1007/s10278-022-00731-7

**Published:** 2022-11-09

**Authors:** Claes Lundström, Martin Lindvall

**Affiliations:** 1 Center for Medical Image Science and Visualization, Linköping University, Linköping, Sweden; 2 Sectra AB, Linköping, Sweden

**Keywords:** Artificial intelligence, Quality assurance, Radiology, Pathology, Validation, Monitoring

## Abstract

The discussion on artificial intelligence (AI) solutions in diagnostic imaging has matured in recent years. The potential value of AI adoption is well established, as are the potential risks associated. Much focus has, rightfully, been on regulatory certification of AI products, with the strong incentive of being an enabling step for the commercial actors. It is, however, becoming evident that regulatory approval is not enough to ensure safe and effective AI usage in the local setting. In other words, care providers need to develop and implement quality assurance (QA) approaches for AI solutions in diagnostic imaging. The domain of AI-specific QA is still in an early development phase. We contribute to this development by describing the current landscape of QA-for-AI approaches in medical imaging, with focus on radiology and pathology. We map the potential quality threats and review the existing QA approaches in relation to those threats. We propose a practical categorization of QA approaches, based on key characteristics corresponding to means, situation, and purpose. The review highlights the heterogeneity of methods and practices relevant for this domain and points to targets for future research efforts.

## Background

There have been massive advances in artificial intelligence (AI) for diagnostic imaging in recent years, with a vast amount of studies showing expert-level performance and many commercial solutions now being available for implementation. The translation of these solutions into actual use in healthcare is, however, still quite limited, a situation that has been described as an implementation chasm [1]. One of the major barriers is how to ensure safety and effectiveness in clinical use, which we will refer to as quality assurance (QA).

Previously, much focus has been given to the model validation by the AI vendor, as this is the initial step to prove that a predictive performance is at a level interesting for healthcare use. It is, however, clear that these validations are not sufficient.

In recent years, the necessity of the care provider doing local validation as a tollgate activity during clinical implementation has become apparent [2–5]. The generalization ability of AI solutions, to retain performance when applied at new institutions, is recognized as a fundamental and severe challenge in the domain. As an illustration, a study investigating performance of three commercial AI solutions for mammography screening found that two of them suffered from generalization issues [6]. While regulatory approval provides a necessary proof point of overall performance, it is also clear that the level of scientific evidence is yet low compared to normal medical standards [7, 8]. The conclusion made from the implementation experiences so far is that the local validations are essential and yet underdeveloped — currently, this is a major impediment for AI adoption [3, 9].

After the initial local validation when the AI solution is in operation, the phase of continuous monitoring follows. The need for continuous monitoring is well established from the AI engineering perspective [10, 11]. Also from a healthcare perspective, the importance of such continuous QA for AI solutions in clinical use has been thoroughly underlined [2, 12, 13], and it is highlighted also from a regulatory standpoint by the Food and Drugs Administration as part of the post-market surveillance [14]. The clinical imaging domain has been deemed particularly challenging for AI monitoring, due to lack of established standards and best practices [2].

An important part of the background to QA for AI is to recognize the many facets that quality encompasses in this context. Zhang et al. [15] differentiate the following quality threats to be tested in relation to machine learning solutions:

- Correctness (predictive performance)
- Model relevance (balanced complexity of model in relation to data)
- Robustness (resilience to perturbations)
- Security (resilience against intentional harm)
- Efficiency (prediction times)
- Fairness (avoiding bias)
- Interpretability (transparency of predictions)
- Privacy (avoiding unauthorized access)

A similar picture of the quality perspectives is given by the ethics guidelines for trustworthy AI from the European Union [16]. Their seven key requirements map nicely to Zhang’s listing of quality threats [17]. The exception is that the EU guidelines emphasize “human agency and oversight,” taking the interpretability aspect one step further. Recent recommendations on trustworthy AI for medical imaging from [18] provide further practical guidance with respect to the quality dimensions.

## Review

The aim with our investigation is to map the QA for AI landscape relevant for the care provider to consider. We do this in two steps. First, we will put the generic quality threats presented above into the diagnostic imaging context. With this as a base, we will then explore the types of QA methods that could be adopted to address the threats in local validation and continuous monitoring settings.

The heterogeneity of aspects potentially relevant to this landscape mapping presents a significant challenge for a traditional systematic review since the scope would quickly become unfeasible. Therefore, a review based on a small set of keywords was deemed inappropriate. Instead, we adopted an exploratory approach. As a starting point, we searched for previous work targeting three types of discussions: clinical implementation of AI in diagnostic imaging, state-of-the-art reviews of AI in diagnostic imaging, and quality assurance of AI (both general and specific for diagnostic imaging). Forward and backward citation chains were then used to expand the set of relevant literature. The elicited listing of the threats and QA method types was refined by the authors across several iterations, and as insights formed, further directed literature searches were made. The review was considered mature once the definitions of threats and method types did not show discordance to the literature, and there were recent literature examples well illustrating the concepts.

### Quality Threats

We will below focus on the quality aspects directly corresponding to value in a health economic sense — essentially that the solutions are safe and effective. Moreover, efficiency in Zhang’s listing refers to inference times, which we consider a minor obstacle for AI medical imaging at this point compared to other aspects. For these reasons, we will not discuss the *security*, *privacy*, or *efficiency* threats further.

*Correctness* is, naturally, at the core of QA for AI in clinical use. A suboptimal correctness will entail suboptimal or adverse effects for some patients. Perfect accuracy is likely to never be achievable. Empirically, the error rate of deep learning models has been shown to follow power-law characteristics with respect to the amount of training data [19, 20], meaning that errors will never vanish by adding more training data.

The *fairness* aspect is getting more and more attention, with AI both introducing a risk of cementing or even aggravating bias, as well as being a potential force to increase objectivity. Fairness threats come in many forms. Illustrative examples include AI training data sets representing only a small and homogeneous part of the world [21], and AI showing a strong capacity to predict race from radiology images which could lead to undetected racial bias [22]. Fairness relates closely to correctness, in the sense that fairness issues translate to predictions having relatively lower precision for a subgroup.

The generalization challenge, to retain performance in new settings, has rightfully been in focus for AI in medical imaging [9]. This quality threat corresponds both to *correctness*, as the impact is predictions of lower precision, and to *model relevance*, as the cause can often be overfitting during training — a model being too tailored for the training data to perform well in new settings. It is also closely connected to *robustness*, where applying an AI model to a new institution’s data can be seen as a “perturbation” of the distribution of the original training data.

**Fig. 1:**
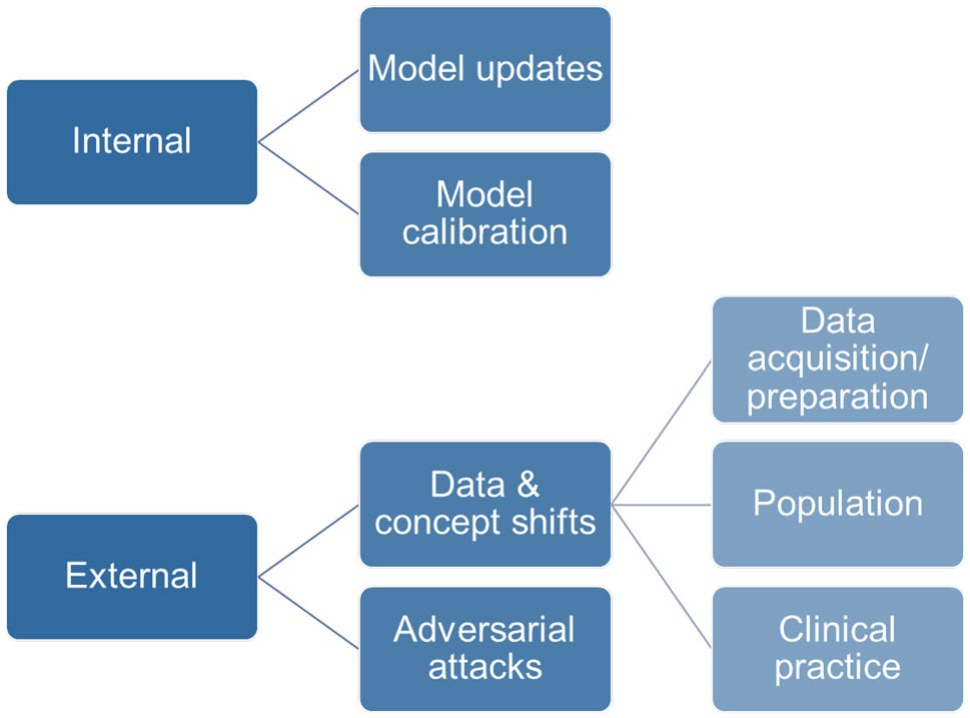
Types of changes to the setting of an AI application that pose quality threats to its robustness

*Robustness* in the sense of being resilient to changes over time is a key aspect that will become increasingly important as the AI solutions pass go from initial deployment to being operational over longer periods. Inspired by the description by Mahadevaiah et al. [23] and Sendak et al. [14], we provide a categorization of change types to consider in Fig. 1.

Internal changes to the model, by deploying a re-trained updated version or by tuning the model in use, are obviously changes that incur risks of decreased quality. The management of these changes is, however, facilitated by the fact that they typically are explicitly planned, discrete events. In contrast, the external factors can be more difficult to detect and act on. Intentionally harmful attacks to the prediction quality is a possibility to consider, but hopefully a very rare situation, whereas shifts in data characteristics are to be expected as a recurring and common challenge. The source can be data acquisition, such as update of the software of a computed tomography scanner entailing a data shift that may affect AI performance [24]. The population being imaged can change, for instance, due to a pandemic or demographic developments, and the clinical practice can cause changes, for instance, due to adjustments in recommended patient pathways [24]. Note that some of these drift effects do not necessarily cause the input data distribution to change, but only the concepts determining how to interpret them [25].

In all cases of data and concept drift, typical errors that could occur would be that the AI model has an incorrectly calibrated operating point that it underreacts to a relevant but previously unseen feature, or overreacts to an irrelevant previously unseen feature.

Finally, *interpretability* is often brought forward as a key component of high-quality AI. Quality threats from this aspect include that poor understanding of prediction rationale may prevent human experts to detect and act on error cases. There are much ongoing research efforts to this end, often under the headline explainable AI (XAI) [26, 27]. Achieving useful interpretability is highly challenging, and critique has been voiced that XAI methods may not be as meaningful as they seem [28]. An important distinction is also the setting when interpretability methods are applied. In the clinical context, explanation methods may be more appropriate in a validation effort of a case batch than for single patients during diagnostic work-up [27, 28].

Another aspect is that interpretability can be crucial for building trust among the human stakeholders, and therefore for the assessment of whether to adopt and use AI solutions. The objective should be balanced trust, as both over-reliance and under-reliance of AI results would hamper their usefulness [29, 30].

### QA Approaches

As seen above, the concept of quality is highly multi-faceted, and the landscape of QA approaches to address quality threats is likewise wide and heterogeneous. An intended contribution of our exploration is to provide a structure for categorizing QA methods for AI in clinical use. Our analysis of the existing body of literature resulted in the following main dimensions emerging:

1. Means: *computation-centric* vs *procedure-centric*
2. Situation: *within diagnostics* vs *separate from diagnostics*
3. Purpose: *identify potential quality issue* vs *act on identified quality issue*

The *means* dimension differentiates between methods that primarily rely on advanced computational analysis and methods that are based on designing clinical production workflows to include QA functions. The computational analysis can be done on the imaging data itself or other data produced during AI processing. In terms of *situation*, it is important to separate the scenario when diagnosticians interrogate the AI model results during the regular review of a single patient from a scenario when cases and their corresponding AI results are scrutinized as a separate QA activity, often in larger batches. We have also identified a need to differentiate the *purpose* of the QA approach, whether it is to monitor for potential issues, or whether the approach also includes acting on identified issues to remedy the issue at hand.

We have identified seven groups of approaches, summarized in Table 1, that we will describe next. The description of each group hinges on a few illustrative examples from previous efforts. In order to make the grouping more valuable as inspiration for future efforts, we have aimed to also map out potential variants not yet explored.

**Table 1:** Overview of the groups of QA-for-AI approaches

**Approach**	**Means**	**Situation**	**Purpose**
Supervised local performance evaluation	Computation	Separate	Identify
Input and output data shift detection	Computation	Separate	Identify
Auxiliary human/AI triage	Computation	Within	Act
Interactive verification	Procedure	Within	Act
Manual spot check review	Procedure	Separate	Identify
Clinical output analysis	Computation	Separate	Identify
Issue-targeted scrutiny	Computation	Separate	Act

#### Supervised Local Performance Evaluation

The first approach to mention with regard to care provider-led QA for AI is local variants of the performance evaluation also done during development and regulatory approval. The main idea is to collect a representative local dataset, establish its ground truth, and check how precise the AI model’s performance is.

The representativeness of the local dataset is of utmost importance. Homeyer et al. [31] propose a systematic analysis of all variability factors having impact on the image data and provide recommendations on what to consider in the case of digital pathology.

Additional insights on evaluation targets are provided by Liu et al. [3], within their proposed framework for medical algorithmic audits. The framework guides the auditor into critical thinking regarding potential algorithmic errors and exploratory testing of their impact in the given clinical context. Liu et al. underline that the quality aspect of fairness should be given much attention, for instance, through different subgroup analyses.

Actively including particularly challenging cases can be an effective way of interrogating performance and provide valuable input on potential failure modes. These can be gathered based on clinical knowledge of pitfalls [3] or generated through intentional corruption or perturbation of the data.

A related possibility is to use synthetic data and/or proxy prediction tasks for the testing, akin to using phantoms in imaging acquisition studies [32]. This may be a way to scale up the testing with limited means, but the risk of not achieving a representative setting must be considered. Evaluations using synthetic data appear particularly useful to make large-scale investigations of potential effects from model changes (the internal changes from Fig. 1).

#### Input and Output Data Shift Detection

Data shift is, as discussed above, an important quality concern and this is a common target for computational methods applied to batches of data as a way to identify potential issues. For a walkthrough of statistical methods for detecting shift, we refer to Feng et al. [12], while we here will focus on the data sources to be monitored.

To detect subtle changes, computational methods can be applied to data in different parts of the processing pipeline. Data shift is, of course, present in the data input to the AI model, and one possibility is to detect data shifts at the input stage. A recent radiology example is the data drift monitoring method for chest X-ray data by Soin et al. [33], taking multi-modal input data into account.

It can, however, be beneficial to analyze data from the intermediate or final stages of the AI model’s processing. One reason is that the dimensionality is much smaller compared to the imaging data input. Another reason is that data shifts on the input side not necessarily affect the AI analysis, whereas a changed distribution on the output side is a clear sign of a new situation where prediction precision may have changed. Output data shift detection is, however, blind to the situation when changes to the input data do affect performance while not affecting the output distribution. An example focusing on output data in the pathology domain is a model-specific shift metric to compare two data collections [34].

A straightforward approach to spotting output data shift is through recurring supervised local validation, as described in the previous subsection, i.e., focusing on the prediction as output data and analyzing shifts in performance.

#### Auxiliary Human/AI Triage

A high-quality AI model will perform as expected for a vast majority of cases. Reverting to manual scrutiny only for a small subset of cases that the AI model is not trained for is typically manageable. Thus, if there were a QA method that could flag whenever the dataset at hand is out of scope for the AI model, much of the AI safety issue would be resolved. This is the allure of triaging methods that could on-the-fly decide to include or not to include an AI solution in the diagnostic workflow. In these approaches to QA in clinical use, note that the application scenario is for a single case as it is being worked up. Moreover, the purpose of triaging is to act on the issue, simply by avoiding the inadequate AI analysis.

A common approach to accomplish such triaging is to analyze the dataset in comparison to the AI model’s training data, using out-of-distribution (OOD) and anomaly detection methods. The OOD area is a very active field of research, also the subarea specifically targeting medical imaging [35]. Uncertainty estimation methods often underpin the approaches in this group. AI methods can be designed to provide estimates of their predictive uncertainty, for instance, through ensemble architectures [36]. Uncertainty can also be elicited from variation induced by perturbations of the input data, so-called test-time augmentation [37, 38].

Another angle to this type of QA approach is to consider the performance of both the human expert and the AI model, and train an auxiliary AI model to determine which workflow path that is likely to be most effective [39]. Such methods are often referred to as learning-to-defer. Recent results point to potential benefits by introducing uncertainty estimations to refine the deferral accuracy also in these approaches [40].

#### Interactive Verification

In clinical imaging, AI solutions often offer possibilities for the diagnostician to verify the result of the analysis during the work-up of the case [41]. Such interactive verification is a type of QA aimed at catching errors and uncertainties before they affect the patient at hand. The result can be presented together with the location(s) in the image most important for the prediction. This allows the human expert to form their own opinion from the image content and serves as an explanation for the AI result. Other XAI methods may also be useful [27, 28]. Apart from catching errors, interactive verification is also the QA subarea where interpretability mainly can be achieved, which is an AI trustworthiness objective in its own right.

A pitfall for AI assistance is if the needed verification work becomes extensive. This reduces any time savings and can even result in the counterproductive situation that AI “assistance” adds to the workload. This challenge is perhaps most articulated for “needle in the haystack” tasks in the gigapixel images in pathology, where a sensitivity level needed to catch a single rare instance may lead to many false positives to work through. Interaction design specifically targeting verification work has been shown to combine high efficiency with high quality control [42, 43]. An example from radiology is an auxiliary QA-specific AI model used to detect discordance between the radiologist’s report and an AI model in an intracranial hemorrhage setting [44].

Interactive verification has a special role to play among QA approaches, since this is where the wider medical knowledge from human experts can be used to address failure modes. Based on systematic mapping of potential issues [3], the verification efforts can be directed to known weaknesses of the AI solution as implemented in the local setting. Conversely, verification for aspects corresponding to AI strengths can be avoided, thereby optimizing the man–machine teamwork [45].

#### Manual Spot Check Review

In many ways, QA approaches for AI can be inspired by, or copied from, approaches used for diagnostic workflows without AI assistance [12]. One way to control and improve diagnostic quality is peer review [46]. In the AI setting, this could be translated to spot checking where the AI analyses for some selected cases are scrutinized by human experts. (Note that in this category, we refer to manual efforts intended to identify issues, whereas further manual drill-down analysis to define appropriate action is part of *issue-targeted analysis* below.)

The spot checking can be organized in various ways. The reviewers can be diagnosticians, technical professionals, or both. The selection can be randomized, weighted towards case types with higher risk of error, or occur ad hoc through instructions to report suspected issues. The review can be a lightweight checkup or part of a more thorough audit program. The audit approach has, for instance, been proposed to tackle the quality threat of algorithmic bias [47], due to its highly complex nature.

#### Clinical Output Analysis

The most important output of any AI-assisted diagnostic workflow is, of course, the resulting conclusions reported to the referring physician. QA-for-AI approaches analyzing the clinical output have the advantage of encompassing the full man–machine pipeline. Issues can be spotted also when they arise in the interplay between diagnosticians and AI. Conversely, a disadvantage is that issues identified may not be relevant for the AI part of the workflow.

One vital type of clinical output analysis is to gather output from the procedure-centric, single-case approaches (*interactive verification* and *manual spot check review*). When studying output across larger batches, issues may emerge that are undetectable at the single-case level. A key example is to register whenever the human expert adjusts or discards the AI result, which we here will refer to as overruling logs.

Statistics in overruling logs can be informative for different QA purposes [2]. Changes can indicate shifts of all types. As the human interaction is an inherent part, one could also detect issues such as variability between diagnosticians, need for training, or need for best practice discussions.

Another type of clinical output analysis is to apply the output data shift detection methods described in a previous subsection, but on the result of the combined human/AI effort rather than on the AI model’s output.

In this category of QA efforts, we also include local validation in the form of systematic clinical trials. Larger efforts of this nature are particularly pertinent when there are greater changes to the clinical workflows due to introducing the AI solution [5].

#### Issue-Targeted Scrutiny

All the QA approaches described above that are efforts separated from the work-up of a single case have one characteristic in common: they are aimed at detecting issues, rather than acting on them. This final category of *issue-targeted scrutiny* gathers all the subsequent efforts to analyze the identified problems and finding remedies to them. The remedies highly depend on the root causes uncovered, but technical improvements such as retraining the model or refining data pipelines are often mentioned [2, 12, 13]. It is, however, important to remember that solutions may also lie in adjusting usage practices, improving the interaction design, or providing additional training.

Another aspect to consider is the risk that the identified potential issue is a false alarm or has negligible effect. For example, this is an inherent difficulty in data shift detection [33]. In general, the more one deploys automated quality issue indicators, the more you need to deal with in terms of drill-down efforts.

### Mapping Threats to QA Approaches

To summarize, Table 2 provides a mapping between the discussed quality threats and the categories of QA approaches presented. The aim is to denote the main threats addressed by each category of methods, even though there may be some benefits also regarding other threats.

**Table 2:** Mapping of QA approaches to quality threats for AI in diagnostic imaging

	**Correctness**	**Model relevance**	**Robustness**	**Fairness**	**Interpretability**
Supervised local performance evaluation	•	•	•	•	
Input and output data shift detection	•		•		
Auxiliary human/AI triage	•	•	•		
Interactive verification	•	•	•		•
Manual spot check review	•	•	•	•	
Clinical output analysis	•	•	•	•	
Issue-targeted scrutiny	•	•	•	•	•

## Discussion

Some overall conclusions can be made from the review regarding current status of QA for AI in diagnostic imaging. There is broad consensus on the need for QA tools and practices in this domain. Validation and continuous monitoring at a local care provider level will be necessary, in order for the care provider to ensure safe usage and tailor an effective complete workflow. There is also consensus on that the area is in its early stages, much yet needs to be developed. On the one hand, one could argue that QA for AI in principle is no different from QA of other medical technology. For instance, the basic regulatory framework is the same. However, many publications in this review point to complexities in AI implementation that are novel to healthcare and require specific consideration. Thus, while many principles and components can be borrowed from traditional QA, new and adjusted approaches are needed, as well as extended skill sets in healthcare organizations.

The review clearly shows that the landscape of QA approaches is heterogeneous. The relevant efforts span from advanced mathematical analyses to applying tacit medical knowledge. Thus, a particular challenge will be to build teams and organizations where professionals of very different expertise can collaborate closely and effectively. It is important to note that while the mapping describes considerations of wide applicability, it does not suggest that all its parts should be implemented for all AI solutions or all care providers. Aspects may be irrelevant or unfeasible to study in the scenario at hand, and there is always an economic trade-off as the execution of the listed approaches can be costly. With respect to the effort level, it has been highlighted that it will be difficult for smaller care providers to adopt diligent AI implementation practices [2]. This means that another area of necessary development is systems and procedures to collaborate across care providers in this domain.

A neighboring area to QA of AI is to use AI for quality control of diagnosticians’ work, as highlighted by Weisberg et al. [48]. While AI as QA for manual work is not in scope for this review, it is difficult to draw firm boundaries between these two areas and there is risk for confusion. Using AI to present potentially missed findings to a diagnostician would by many be considered a standard AI workflow, whereas Weisberg et al. argue that it is beneficial to think of it as a QA workflow.

In this area, there are many questions where appropriate answers are lacking today. For example, for industry and technical researchers: What QA tools are most prioritized to develop? Can we train AI models in ways that make subsequent QA easier? For care providers: What is an appropriate effort level for local validation and monitoring? What happens to the cost–benefit balance when adding QA efforts?

The exploratory approach to this review comes with important limitations. In relation to a traditional systematic review, there is a higher risk that relevant work has been overlooked. Going forward, we expect and wish that our mapping is challenged, expanded, and refined. Nevertheless, we believe that the main traits of the proposed mapping will prove to be stable and, hopefully, useful for efforts to implement AI for diagnostic imaging safely and effectively.

The development of QA for AI in diagnostic imaging is likely to benefit from established knowledge and practices in neighboring fields. The MLops concepts [10], for instance, are more developed in industrial applications. Established knowledge in the domain of resilience engineering may also prove useful [49]: Change is often incremental and unanticipated, and building resilience translates to establishing multiple, overlapping approaches that can absorb and adapt to those changes.

Thus, the objective should not be to optimize a single QA tool, but rather to establish sound QA practices using a comprehensive set of tools covering different aspects and scenarios. We argue that such a panorama of indications from different QA approaches also is a good way for organizations and professionals in healthcare to arrive at an appropriate, balanced level of trust in AI.

## Summary

To ensure safe and effective AI usage in diagnostic imaging, care providers need to develop and implement local QA approaches. While this need is undisputed, the domain of AI-specific QA is still in an early development phase and the mapping presented aims to assist care providers, researchers, and developers in navigating the heterogeneous landscape.
